# DNA Origami Disguises Herpes Simplex Virus 1 Particles and Controls Their Virulence

**DOI:** 10.3390/molecules27217162

**Published:** 2022-10-23

**Authors:** Raina M. Borum, Avery E. Lin, Xiangyi Dong, Mingxuan Kai, Yi Chen

**Affiliations:** Department of NanoEngineering, University of California, San Diego, CA 92093, USA

**Keywords:** DNA origami, herpes simplex virus, folic acid, targeted delivery

## Abstract

DNA nanostructures are well-established vectors for packaging diversified payloads for targeted cellular delivery. Here, DNA origami rectangular sheets were combined with Herpes Simplex Virus 1 (HSV1) capsids to demonstrate surface coverage of the particle via electrostatic interactions. The optimized origami:HSV1 molar ratios led to characteristic packaging geometries ranging from dispersed “HSV1 pockets” to agglomerated “HSV1 sleeves”. “Pockets” were disguised from cells in HeLa and B16F10 cells and were 44.2% less infective than naked HSV1 particles. However, the pockets were 117% more infective than naked HSV1 particles when the origami sheets were coated with folic acid. We observed infectivity from naked origami, but they are 99.1% less infective with respect to HSV1 and 99.6% less infective with respect to the pocket complexes. This work suggests that DNA origami can selectively modulate virus infectivity.

## 1. Introduction

The precise presentation of biomolecules between the surfaces of cells and pathogens drives their interactions. Bacteria, viruses, and other foreign bodies present characteristic proteins to facilitate uptake and infection [1,2,3,4,5,6,7]. Tailored mimicry of this molecular dialogue may offer several advances in synthetic biology. First, details of pathogenicity may be better clarified and emulated. More importantly, controllable pathogenicity may be achieved [8,9] for improved delivery of therapeutically engineered viruses [10] such as oncolytic viruses for cancer therapy [11,12], which are limited by neutralizing antibodies from the host, premature degradation, and liver clearance pathways [13,14,15].

The ability to mask native surface proteins on a virus while presenting a desired class of proteins can disguise the virus and modify its uptake. This approach requires a biocompatible material that can organize the desired proteins at a controllable density while maintaining coverage over the original surface. Cell membrane coating technologies are popular for the disguise and delivery of foreign entities into cells but suffer from coating inefficiencies during fabrication, low-yielding purification from uncoated entities [16], and inflammation with collateral immunogenicity [17]. Polymeric and silica coatings have been proposed, but both materials have minimal control over peptide or protein decoration for cell uptake; they are also toxic towards immune cells via oxidative stress and pro-inflammatory activation while lacking immunostimulatory stealth [18,19,20]. 

DNA nanotechnology uses programmed strands that complimentarily self-assemble into a coherent structure via base-pair specificity. DNA offers an unrivaled ability to organize molecules with single nanometer precision [21,22], fabricate structures with high yield [23,24], and maintain integrity in vivo while delivering therapeutic agents [25,26,27]. Some reported DNA structures have demonstrated low immunogenicity in vivo [27]. DNA nanostructures can be fabricated in several ways including “DNA origami” where small “staple strands” use complimentary forces to fold one long “scaffold strand” into the programmed shape. An alternative is “ODN or oligonucleotide approach” where short oligonucleotides self-assemble into the structure [28].

There are many compelling reports on combining DNA nanostructures with viruses. DNA origami rectangles have been loaded with cowpea chlorotic mosaic virus (CCMV) capsid proteins for increased transfection into cells [29]. DNA “nano-stars” have demonstrated robust avidity to dengue virus (DENV) by precisely organizing aptamers in a pattern identical to the complimentary DENV glycoprotein distribution to mute their infectivity [30]. Elegant icosahedral DNA structures have demonstrated complete entrapment and neutralization of hepatitis B and adeno-associated viruses in human cells [31]. A next step in the field is selective viral infectivity.

**Figure 1:**
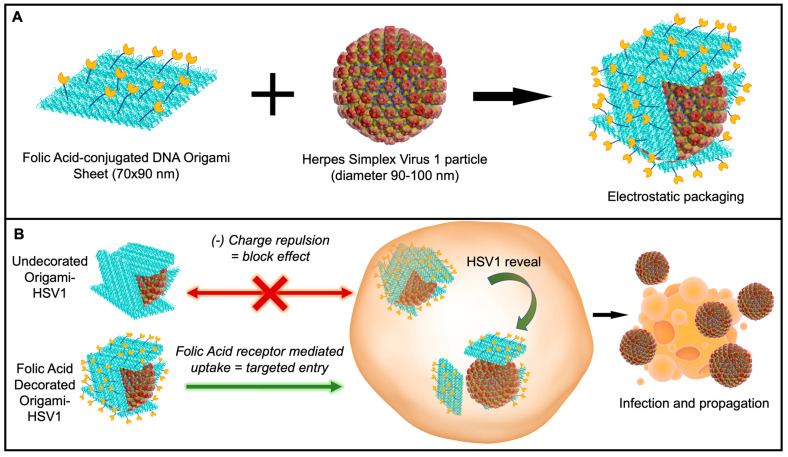
Schematic of Disguised HSV1 through DNA Origami. (**A**) Wrapping HSV1 in folic acid (yellow)-adorned DNA origami (blue). (**B**) In vitro delivery experiments on the (1) disguise of HSV1 and (2) targeted initial uptake into folate-receptor positive cell surfaces.

Here, we report the ability to disguise herpes simplex virus 1 particles with DNA origami nanosheets. The origami sheet carries a net negative charge from the phosphate backbones of the DNA to cover the positively charged HSV1 capsid proteins via electrostatic interactions [32]. We explore and report this packaging mechanism with origami sheets containing overhanging strands that anchor the targeting ligand folic acid on the outer surface, as biomarker receptors are a promising avenue in targeted nanomedicine (Figure 1A) [33,34,35,36]. This folic acid decoration efficiently delivers the viral HSV1 particle cargo to folic acid receptor-positive cell surfaces via the DNA origami (Figure 1B). Without this decoration, charge repulsion between the origami coating and the cell membranes prevents HSV1 entry. This work describes an applied nanomaterial that can modulate delivery of engineered oncolytic viruses; HSV1 is a fundamental backbone for talimogene laherparepvec (T-VEC)—the first FDA-approved oncolytic virus to combat Stage 3 and Stage 4 melanomas and lung cancers [12].

## 2. Materials

The materials for this research include DNA Origami and ssDNA strands (Integrated DNA Technologies (IDT), Coralville, IA, USA), M13mp18 scaffold strand (Guild Biosciences, Dublin, OH, USA), PEG-maleimide-folic acid (NanoCS, New York, NY, USA) Monkey Vero-propagated Herpes Simplex Virus 1 particles (Bio-Rad, MPP010, Hercules, CA, USA), HSV1/2 Major Capsid Protein ICP5 Primary Antibody (Santa Cruz Biotechnology, Dallas TX, USA), Goat anti-mouse IgG Horseradish Peroxidase (BioLegend, San Diego, CA, USA), Azure Radiance Q Substrate (Azure Biosystems, Dublin, CA, USA). HeLa and B16F10 cells were generously gifted by Professor Liangfang Zhang’s Nanomedicine Laboratory (UC San Diego, San Diego, CA, USA).

## 3. Methods

### 3.1. DNA Origami

Plated DNA strands were first centrifuged, then aliquoted to 100 µM with MilliQ water. Strands were then collected to add with the M13mp18. Strands were then mixed at a 1:10 M13mp18 to staple strand molar concentration in 1× TAEMg (Tris-base Acetic acid, EDTA, Mg) buffer. If Folic Acid strands were included in the sample, the strands were also incubated with the folic-acid DNA strands at a 1:10:10 M13mp18:staple:folic acid strand molar ratio. The strand mixtures were first heated to 90 °C for one minute before 3 h cooling to 4 °C. If not immediately used, origamis were stored at 4C.

### 3.2. DNA-Folic Acid Conjugation

Thiolated ssDNA strands (SH-ssDNA) were also ordered from IDT. In summary, the ssDNA-SH was incubated with tris (2-carboxyethyl)phosphine (TCEP) at a 1:10 molar ratio in 50× TAE (Tris-base, Acetic acid, EDTA) for 6 h room temperature, in order to reduce disulfide bonds from the thiol-tagged ssDNA. Maleimide-PEG-Folic Acid was then added to the reaction and given overnight to link to the strands at room temperature. The conjugation reaction was halted and purified by letting the sample run through a spinning dialysis (3000MWCO) tube in a 2 L beaker with MilliQ Water overnight. Conjugation yield was verified through a 20% Urea Denature PAGE.

### 3.3. HSV1-DNA Origami Assembly

HSV1 Particles were obtained from Bio-Rad. For ratio experiments, the HSV1 particles were previously diluted in 1× Phosphate Buffer (1xPBS, pH 7.5). At the specified ratios, HSV1 particles and the pre-made DNA Origami were mixed together in 1.5 M NaCl concentration, diluted as necessary in 1× TAEMg Buffer. After pipetting to promote even mixture, the complexes were allowed 2 h to react at 4 °C. The complexes were either used in 1% Agarose Gel Electrophoresis for mobility shift assaying, directly added to freshly cleaved mica substrates for Atomic Force Microscopy, or directly added with DMEM for cell related experiments.

### 3.4. Complex Characterization

For all complex characterization methods, every sample, (i.e., DNA Origami, naked HSV1 capsid particles, and the complexes at different ratios) were characterized under the same reaction solvent conditions, (1.5 M NaCl, 1xTAEMg buffer) throughout.

Agarose Gel Electrophoresis Mobility Shift Assay (AMSA) AMSA was implemented using a 1% Agarose/1xTAEMg gel. The gel was immersed in 1xTAEMg as the running buffer. The gel was stained with 0.5 μg/mL Ethidium Bromide. After 20 μL of each sample was loaded into each well in the gel, the gel was run at 90 V (constant voltage) for one hour. To prevent heat-denaturing of the DNA Origami higher order structure from electrophoresis adverse heating effects, the gel electrophoresis box was covered and surrounded by dry ice pellets. After the run, the gel was exposed to ultraviolet light for imaging.

Dynamic Light Scattering (DLS) 100 μL of each sample was loaded into a microvolume cuvette, and all DLS measurements were done on a Malvern Zetasizer Nano ZS.

Atomic Force Microscopy (AFM) AFM was performed under room temperature dry conditions. Then, 5 μL of the sample was drop casted onto a freshly cleaved mica surface and incubated at room temperature for several minutes. The substrate was washed with 2 mM Mg (Ac)_2_ solution and dried by compressed air.

### 3.5. Cell Cultures

HeLa cervical cancer cells and B16F10 mouse melanoma cells were cultured in Dulbecco’s Modified Eagle Medium (DMEM) with 10% Fetal Bovine Serum, 1% Penicillin-Streptomycin at 37 °C at 5% CO_2_, and were permitted at least 3 passages before use in experiments.

### 3.6. Viral Plaque Assay

300,000-cell monolayers were first seeded into 6-well plates. After reaching 90–100% confluency, media was decanted and the cells were washed once in ice cold 1× Phosphate Buffer Saline (PBS) before infected with HSV1, DNA Origami, or HSV1-DNA Origami complexes for 2 h at 37 °C, 5% CO_2_. The plates were gently rocked every 30 min to promote even distribution of inoculum. Inoculum was then aspirated and the cells were overlaid with 0.3% Agarose/DMEM and provided 4–6 days for incubation. ImageJ particle analysis was used for plaque counting. Images for processing were converted to 8-bit type before threshold adjustments were held constant for every sample. These plaque formation in the images were then counted through the ImageJ particle analysis tool (Figure S7). Our statistical analysis utilized Welch’s *t*-test, which assumes unequal variance between samples.

Infectivity rate is generally quantified as Plaque Formation Units per volume (*PFU*/mL). This rate is found by relativizing (*P*) plaque number by (*D*) dilution factor of the pathogen and (*V*) total inoculum volume [37] as described below as Formula (1):

(1) $$\frac{PFU}{ml}=\frac{P}{D*V}$$

For the complexes, the infectivity was calculated using Formula (2):

(2) $$\frac{PFU}{ml}=\frac{P}{\phi *V}$$

(3) $$where\;\phi =\frac{\left(D_{Origami}\right)\left(D_{HSV1}\right)}{\left(D_{Origami}\right)+\left(D_{HSV1}\right)}$$

where this formula is described in the discussion section with derivation specified in the supporting information.

### 3.7. In vitro Structural Stability Experiments

All dilutions and packing ratios were replicated as identical to the viral plaque assay experiments. In summary, origami, origami-Folic acid, and HSV1-packed complexes of both origami cases were incubated in same FBS and antibiotic content in DMEM at 37 °C for 2 h, then further analyzed through 1% Agarose Gel Electrophoresis. As a control, these were compared to complexes identically prepared, yet incubated at 4 °C and in extra 1xTAEMg as opposed to the cell culture media.

### 3.8. Western Blotting

1,000,000 cells were seeded into individual T-25 flasks and were provided several days to reach 85–90% confluency. Flasks were then inoculated with HSV1 and Origami-HSV1 complexes and overlaid with sufficient media for one week (a total volume of 4 mL). The media was collected after the infection period and then spun down at 1000× g for 15 min. The top 75% of this supernatant was extracted and mixed with reducing SDS loading buffer (50 mM Tris-HCl pH 6.8, 2% SDS, 10% glycerol, 1% 2-mercaptoethanol, 12.5 mM EDTA, 0.02% bromophenol blue). Samples were run in an SDS Bis-Tris gel at 165 V for 45 min, then transferred to a nitrocellulose membrane at 15 V for 30 min. Membranes were blocked in 5% milk/0.1% PBST for one hour, incubated with HSV1 ICP5 Major Capsid Protein at a 1:1000 ratio in Milk/PBST overnight at 4 °C, then incubated for 2 h with goat anti-mouse IgG-Horseradish Peroxidase at a 1:1000 ratio for 2 h rocking at room temperature. Membrane was then rinsed with deionized water before reacted with Azure Radiance Q Chemiluminescent substrate for 5 min. Autoradiography films were exposed to the membrane for ten seconds before image processing.

## 4. Results and Discussion

**Figure 2:**
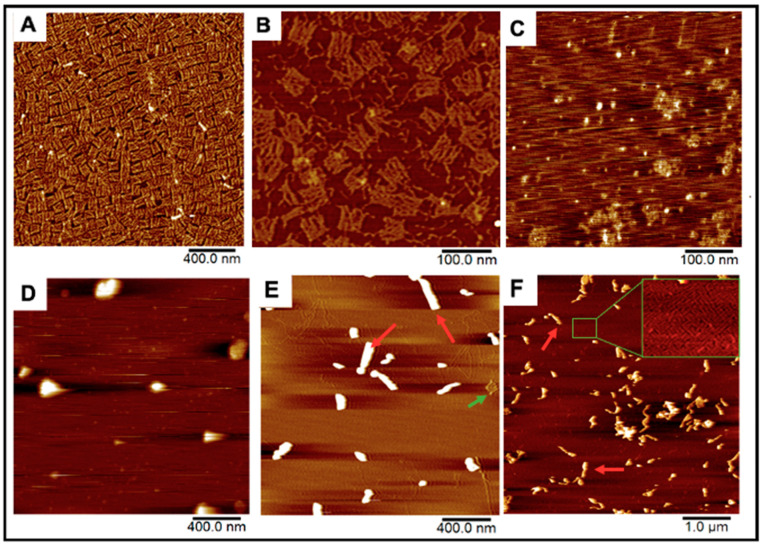
AFM Images of our DNA origami, HSV1 particles, and the resulting complexes after mixing. (**A**,**B**) Origami and folate-decorated origami sheets. Salt bi-products from formed origami were observed as seen in (**B**). (**C**) HSV1 particles (**D**) Origami:HSV1 “pockets” established at a 2:1 Origami:HSV1 molar ratio (**E**) 20:1 ratio “sleeves” and (**F**) 50:1 ratio “sleeves” where many excess sheets are left unbound. Green arrows show unbound sheets in the packing reactions while red arrows show products of the HSV1-origami “sleeve” complexes.

DNA Origami Design. The DNA Origami sheet design was adopted from one of Rothemund’s original DNA rectangle designs [24]. These sheets were first fabricated by mixing 235 staple strands that fold a scaffold strand into 70 × 90 nm rectangles by hybridizing with multiple regions on the scaffold and each other [24]. The scaffold strand is M13mp18 ssDNA, a bacteriophage genome strand that is conventionally used for most origami scaffolds due to its extensive length (7413 nucleotides) [24,28]. Rectangles were successfully formed after annealing all strands together from 90 °C to 4 °C in the span of three hours (Figure 2A). We further installed 12 “overhanging” polyA strands that tether covalently conjugated folic-acid polyT strands for cell-targeting demonstrations (Figure 1, Figure 2B and Figure S1).

DNA Origami-HSV1 Particle Packing. These origami rectangles were then mixed with commercially prepared HSV1 capsid particles from 2:1 to 50:1 molar ratios, where the HSV1 particles previously had their envelope and glycoproteins removed to expose the positive charged capsid by the manufacturer (Figure 2C). The charge-mediated union between the origami and HSV1 particles consistently resulted in specific packing morphologies. The comparable dimensions between the sheets (70 × 90 nm) and the HSV1 capsids (90–100 nm in diameter) allowed us to hypothesize that 2:1 and 3:1 origami:HSV1 molar ratios can sufficiently cover the virus. Under these proportions, the particles were wrapped in dispersed, rounded, “pocket-like” structures as seen via atomic force microscopy (AFM) (Figure 2D). However, as we decreased HSV1 content so that the ratio shifted towards 10:1, 20:1 and 30:1, the packaged complexes assembled into elongated or “sleeve-like” structures, where capsid aggregates were packaged into many origami sheets at once with minimal unbound sheets remaining in the reaction (Figure 2E). Yet, at higher 40:1 and 50:1 ratios, these sleeve structures were sustained while many unbound origamis were observed, suggesting that a critical packing ratio between the HSV1 particles and DNA sheets had been surpassed (Figure 2F).

**Figure 3:**
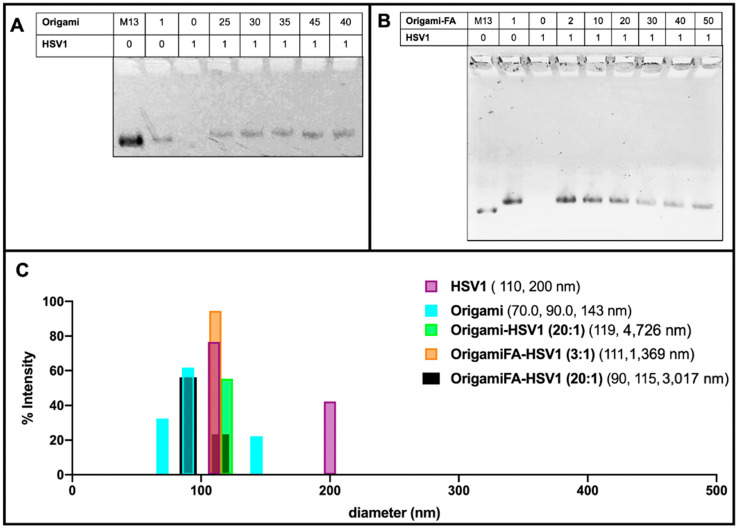
Electrostatically HSV1 particle-loaded DNA Origami. (**A**) 1% Agarose Mobility Shift Assay (AMSA) analysis of HSV1-loaded origami shows a sudden change in mobility between 35:1 and 40:1 origami:HSV1 ratios from otherwise decreased mobility, suggesting the critical packing ratio and (**B**) Folic-Acid adorned origami similarly loaded with HSV1 particles, where 20:1 is the demonstrated critical packing ratio. Both (**A**,**B**) also show a decreased mobility from the free M13mp18 scaffold strand to the fully annealed DNA Origami in the first two left lanes of both gels. (**C**) Dynamic Light Scattering validation of HSV1 particle and DNA Origami size dimensions where occasional dimerized configurations were observed. Data also confirms the complex package sizes at a 2:1 ratio origami-HSV1 and 3:1 ratio of origami Folic Acid to HSV1 complexes. No leftover origamis are observed, but agglomeration is noted.

These packing behaviors were further validated with 1% agarose gel electrophoresis mobility shift assays (AMSA). There was no visible band from the lane loaded with naked HSV1 particles because their positive charge prevented them from traveling along the same electrophoretic direction as origami-related samples. (Figure 3A,B). On the other hand, the origami band displayed lower mobilities as the HSV1 particles were packaged within the origami structures. As previously realized in the AFM micrographs, AMSA confirmed a critical ratio between 35:1 and 40:1 provided the sudden lighter mobility of the 40:1 and 45:1 packing ratios with respect to the other loaded ratios (Figure 3A and Figure S3).

Dynamic Light Scattering measurements further validated this capsid coating behavior. When complexed at a 2:1 origami:HSV1 ratio, 55.4% of size dimensions were 98.51 nm in diameter with 44.3% 308 nm in diameter. These measured sizes are comparable to the dimensions of single-particle and dimerized aggregates. However, we chose to use the DLS data of higher packing ratios as a supplement provided that micron range dimensions—as was seen in the sleeves—lead to less accurate scattering properties (Figure 3B,C and Figure S2A–D).

Dynamic light scattering also confirmed the behavior of the HSV1 capsids alone. If the capsid particles were otherwise not disperse in the solvent, as is seen with the detected 110 nm size measurement, which is both expected of the capsids and the majority of the detected size of the sample, there was occasional dimerization (as is seen with 200 nm detected sizes), but no agglomeration or submicron to micron clustering was observed. This implies that when the capsids are packed into larger sized aggregates by the origami, it is not due to their propensity to aggregate alone. 

While Folic Acid-functionalized origamis displayed loading capabilities like the unfunctionalized origamis, AMSA experiments clarified that the critical packing ratio shifted from 35:1 to near 20:1 (Figure 3B). We believe the extra positive charge from the folic acid led to a diminished efficiency in electrostatic packaging between the negatively-charged DNA sheets and the positively-charged virus capsid [38]. Regardless of the sudden change in band mobility after the 20:1 ratio, the bands continued to decrease in intensity even as 30:1, 40:1 and 50:1 ratios were explored. This may be due to a wider distribution of packing morphologies such as a combination of pockets, longer sleeves, and shorter sleeves (Figure 3B and Figure S4). Moreover, the decreased band intensity is caused by the majority of the loaded sample remaining in the well rather than traveling down the gel matrix; there are thus complexes of larger sizes under these ratios. This distribution behavior was further validated through DLS and confirmed the increased polydispersity index (PDI) values of 0.81, 0.96 and 1.00 for 30:1, 40:1 and 50:1 ratios, respectively. The PDI values for lower ratios ranged between 0.55 to 0.71.

The AFM images at both low and high magnification, in combination with the DLS data presented in Figure 2 and Figure 3, indicate that higher ratios of origami to HSV1 particles promote linear, “sleeve-like” assemblies. This conformation is most likely due to patchy aggregation before full outer coverage: The higher amount of available origami sheets allows some of them to bind in between particles as electrostatic patches first [39], bridging the particles together while residual sheets cover remaining exposed and positive charged regions on the capsid particles’ surfaces [40,41]. The presence of sleeves is made possible due to more available free origami sheets, but it is not necessarily the preferred assembly, as is exemplified by the high PDI values from DLS and the persistent presence of pockets at these higher ratios.

**Figure 4:**
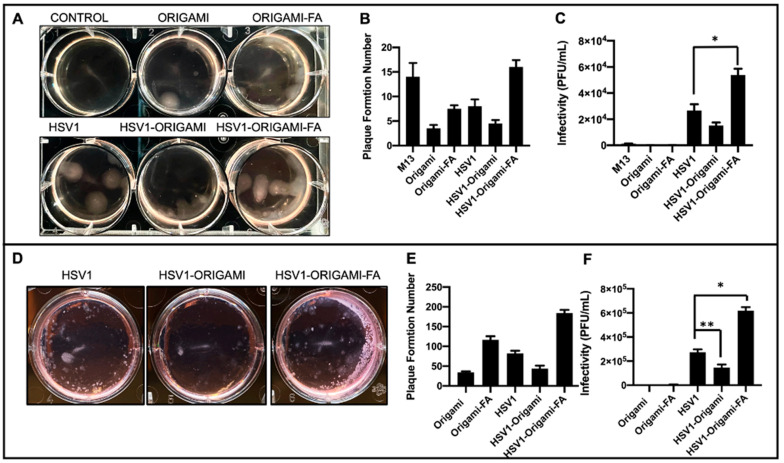
In vitro analysis of Origami-HSV1 complexes. (**A**) Plaque assay of HeLa cells showing HSV1 in origami-Folic Acid led to heightened plaque formation above HSV1, but also showed muted plaque formation for HSV1 wrapped in bare origami. (**B**) Quantified plaque formation numbers from those observed in panel A as is described in the methods section and Figure S6. (**C**) The infectivity rates (Plaque Formation Unit/mL) shows that DNA Origami plaque leads to negligible infectivity relative to HSV1 and complexes, and that HSV1 wrapped in origami-Folic Acid is the most infective above native HSV1 (* denotes Welch’s unpaired *t*-test, *p* = 0.0293). (**D**) Plaque Assays of Mouse Melanoma B16F10 show heightened plaque formation from HSV1 in folic acid decorated origami complexes alongside decreased plaque formation from HSV1 in bare origami as compared to HSV1 alone. (**E**) Quantified plaque formation numbers of infected B16F10. (**F**) Resulting infectivity rate (Plaque Formation Unit/mL) of B16F10 outlining comparable behavior as those results from the HeLa plaque assays (* denotes Welch’s unpaired *t*-test, *p* = 0.0065, ** denotes Welch’s unpaired *t*-test *p* = 0.0370). Error bars are the standard deviation.

Infectivity Analysis in vitro. After establishing these structural and packing properties, we studied the virulence of origami-HSV1 and Folic-Acid-Origami-HSV1 complexes using the 3:1 origami:HSV1 molar packing ratio. Plaque assays were implemented here because they are the gold standard for titering and characterizing infectivity rate of pathogens [37,42,43,44]. To summarize the process, cell monolayers are infected with the pathogen and then cleaned and further fixed in agarose-media to localize plaque formations. The epicenters of infections are within the monolayers and identified as plaque [37]. Here, HeLa cells were used as a model cell line due to their density in folic acid receptors [45]. When HeLa cultures were inoculated, there was a 46.8% heightened plaque formation from folate-decorated HSV1-origami pockets above naked HSV1 particles alone. Surprisingly, there was a 42.1% less plaque formation when the HSV1 particles were masked in undecorated origami (Figure 4A,B). We believe that this muted formation is due to negative charge repulsion between the cell membranes and the phosphate backbones of the bare origami surfaces [29,46].

There was plaque formation in the HeLa cells when they were inoculated with the naked origami sheets—either bare or further decorated with the folic acid. (Figure 4A,B). Yet, both cases resulted in fewer plaque formations than HeLa cells inoculated with the naked “scaffold strand,” M13mp18 (Figure 4B and Figure S6). Prior work has shown that foreign M13mp18 strands can become internalized and persist in eukaryotic cell lines [47,48,49]. Our data show that the M13mp18 strands generated 300% more plaque than the bare origami and 89.3% more than folate-decorated origami due to suppressed exposure of the strand from hierarchical folding with the staple strands. While both origami designs produced plaque, the folate-functionalized origami generated an average of 121% more plaque than the bare origami suggesting a forced uptake of the origami through folic acid reception.

However, plaque formation numbers alone are an incomplete outline on the virulence of the origami, HSV1, and HSV1-Origami complexes. To elucidate this, the infectivity rate is generally quantified as Plaque Formation Units per volume (PFU/mL). This rate is found by relativizing (P) plaque number by (D) dilution factor of the pathogen and (V) total inoculum volume [37]. This applies directly to the HeLa inoculated with the origami sheets, Folate-decorated origami sheets, and naked HSV1 particles (Formula (1)).

However, because the origami and HSV1 particles are simultaneously administered at differing dilution factors when the packed complex is the inoculum, a parallel function is necessary to compensate for both entities. (Formula (2)) shows the justification and derivation of parallel function further detailed in the Supplementary Information). 

These results indicate that the plaque-forming origami sheets did not contribute to infectivity rates comparable to the native HSV1 particles and the complexes—either bare or further adorned with folic acid (Figure 4C). In fact, naked origami was at least 99.7% less infective than complexes and 99.3% less infective than naked HSV1. This claim is further supported in that the naked origamis were inoculated at the same dilution factors as the origamis involved in the complex inoculums.

The infectivity rates of these complexes against B16F10 mouse melanoma cells were also tested under identical conditions as those with the HeLa cells. Because the HSV1 particles are natively propagated in Vero cells and are therefore ape-derived, we hypothesized it would be increasingly difficult for them to establish initial infectivity in a mouse cell line [50] unless coerced inside by folic-acid mediated uptake. While HeLa and B16F10 are from different species, both are folic acid receptor-positive [51]. Indeed, B16F10 infection experiments showed similar plaque formation behaviors as observed during the HeLa experiments, but the infectivity of the folic acid-decorated origami-HSV1 pockets led to a 127% increase over the native HSV1 (Figure 4D–F). The B16F10 line formed at least 30 more plaque formations than HeLa, but their sizes were significantly smaller than those in the HeLa line (Figure 4A,D). The smaller plaque sizes are in fact due to infection attenuation from the virus [52] likely because the HSV1 is infecting a species foreign to its own origin (Figure 4D).

**Figure 5:**
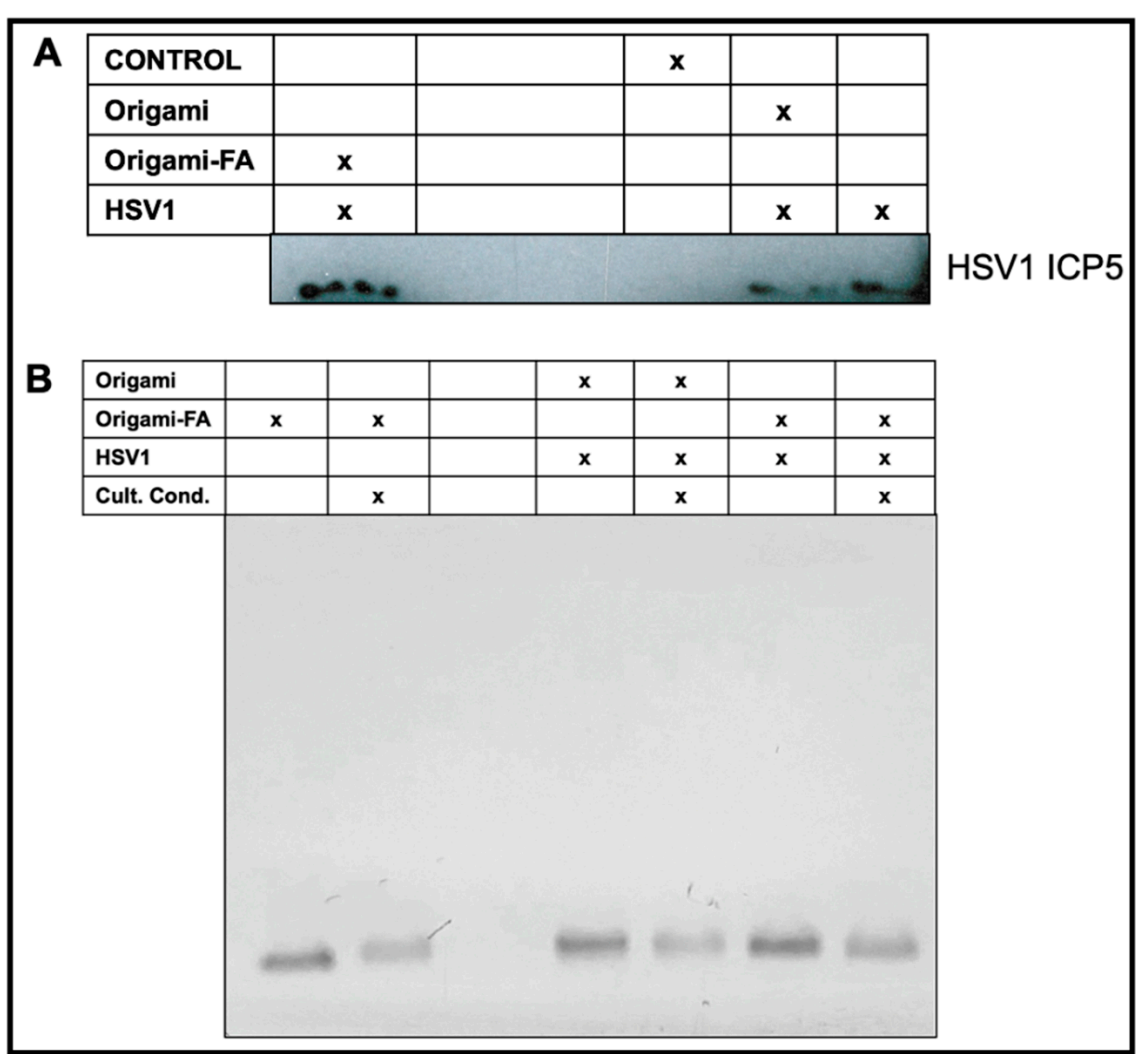
Validated HSV1 delivery and packaging stability of the Origami-HSV1 complexes in vitro. (**A**) Western Blot characterization on produced ICP5 HSV1 Major Capsid after HSV1 infection based on delivery or masking method. (**B**) Validated stability of the Folate-adorned Origami-HSV1 complexes in vitro under cell culture conditions identical to the conditions during the plaque assay inoculum stage. The corresponding table legends for (**A**,**B**) indicate the present entities and conditions for each sample in each lane.

Validation of HSV1-Origami Complex stabilities in vitro. We next tested if the increased plaque from the HSV1-Origami-Folic Acid complexes was due to their short stability in vitro, i.e., a mere accumulation of the origami and HSV1 plaque formations from full-dismantlement in cell culture conditions. There are three approaches to resolve this question. First, we implemented Western blotting to directly monitor changes in the concentration of produced HSV1 ICP5—the major capsid protein. This is an accurate method to measure HSV1 based on delivery methods because the origami would not contribute to the ICP5 band (wrapped in bare or decorated origami, or administered completely naked). The Western Blot confirmed that the folate-adorned origami wrapped HSV1 led to the most ICP5 protein even over naked HSV1 particles (Figure 5A).

Second, AMSA analysis was implemented on the complexes to examine their structural stability under cell culture conditions. Incubation of DNA origami in standard cell culture conditions (10% FBS, Dulbecco’s Modified Eagle Medium, 1% Pen-Step under 37 °C at 5% CO_2_) led to (1) denaturation of the structure and (2) digestion of the structural DNA due to nuclease activity and diminished cationic stability [53,54]. We therefore incubated origami and origami-HSV1 complexes under these conditions while strictly matching incubation time periods and dilutions as was performed in the plaque assays. We then compared these samples to those left at the bench under the same time. There was no mobility shift when the origami was loaded with HSV1 and then incubated under cell culture conditions (Figure 5B). While the band intensity slightly decreased suggesting some digestion activity, the remaining origami structures do maintain their binding to the HSV1 particles due to the unshifted band. If the band lowered to its original level when unloaded, then this would suggest that the free-floating origami is exposed and infective to the cells on their own.

Each inoculum led to consistent plaque numbers, and thus we implemented mathematical predictions on the resultant infectivity of these complexes including if the origami completely detached from the HSV1 particles or completely stuck with them during administration in vitro. If the complexes dissociated before they reached the cells for infection, then the plaque from the complexes would have resulted in numbers similar to the sum of the plaques from the origamis and HSV1 particles alone. However, if they remained completely intact prior to reaching the cell, then the resultant plaque from the complex might resemble plaque formed but with the plaque from the origamis alone subtracted (Figure S8). When we modeled both of these cases and compared them with the empirical results, neither of the complex infectivity rates compared with the predicted detachment cases. On the other hand, they resulted in heightened infectivity numbers than those in the fully attached case but it is crucial to note that we did not account for the continuous production of the HSV1 particles—leading to more infectivity—when the complexes are fully attached. The results suggest that the HSV1-Origami complexes maintained their packing integrity when employed for infections against the cell lines, and that enhanced infectivity form the folic acid-adorned Origami-HSV1 pockets is due to the increased efficiency of their uptake.

Here, for the first time, HSV1 particles were wrapped in DNA origami and evaluated for controllable viral delivery. Inspired by past demonstrations on DNA origami’s electrostatic affinity towards positively charged viral plant capsid particles, we corroborated the translatability of this binding interaction with a larger mammalian viral capsid particle [29]. When the particles are wrapped in bare origami sheets, their recognition and uptake into cells are noticeably diminished by a 44.2% decreased infectivity rate. This muted infectivity further supports previous reports on DNA origami’s potential to inhibit viral infections by structurally covering and thereby trapping virus cores [30,31]. On the other hand, when wrapped in origami that is outwardly functionalized with folic acid, folate-receptor mediated uptake allowed the viruses to have increased HSV1 protein production and 117% heightened infectivity. This therefore reveals unique and potentially therapeutic value in the context of engineered, antitumor viral infections. Plaque assays were used to monitor the infectivity of the Origami-HSV1 complexes and the origami sheets themselves. While origami sheets displayed plaque forming and infective behavior, we validated that (1) their infectivity is negligible relative to native HSV1 and (2) they maintained packing integrity in vitro. Thus, the origami sheets did not individually contribute to the enhanced infectivity of the HSV1-wrapped origami complex in folic acid. Future work may investigate the administration of these complexes during longer inoculation times both in vitro and in vivo to further elucidate their stability. Different packaging mechanisms besides electrostatic binding can be explored. Perhaps most importantly, these results encourage future research on DNA nanotechnology—specifically origami—in a system where pathogenicity is more rigorously examined. Although more recent reports continue to demonstrate the nontoxic and low immunogenicity of DNA origami when administered in vivo [27,55], more stringent characterizations on origami geometries and potential adverse effects should be investigated. This will be a large stride in the global effort to introduce DNA Origami—and DNA Nanotechnology altogether—into serious consideration for therapeutic translatability.

## 5. Associated Content

Proof and derivation for complex infectivity. Bright field confocal micrographs taken 12 h after HeLa is inoculated. DNA strand sequences. Twenty percent Denature gel characterizing the ssDNA-Folic Acid conjugation efficiency. M13mp18 plaque assay results. ImageJ plaque assay analysis. Modeled predicted versus empirical infectivity rates (PFU/mL) of HSV1 and HSV1-Origami complexes, either assuming full-detachment during inoculum and full-attachment during inoculum period. Sequenced staple strands for DNA Origami. Supplementary Dynamic Light Scattering data on complexes based on packing ratio.

## Data Availability

Not applicable.

## Supplementary Materials

The following supporting information can be downloaded at: https://www.mdpi.com/article/10.3390/molecules27217162/s1, Figure S1: 20% Urea Denature PAGE on Folic Acid-DNA conjugation. Figure S2: Supplementary DLS measurements of our origami:HSV1 complexes based on molar packing ratio. Figure S3: Further 1% AGE validation of Origami-HSV1 loading depending on molar ratios. Figure S4: Low Magnification Atomic Force Micrographs of DNA Origami-HSV1 Complexes Figure S5: HeLa cells 24 h post infection. Figure S6: Plaque Assay result of HeLa inoculated with naked M13 strands at the same concentration as inoculated origami. Figure S7: Plaque Assay Plaque counting using ImageJ. Figure S8: Predicted infectivity rates based on complex stability or instability in vitro, as compared with empirical results. DNA Origami staple strand sequences.

## Abbreviations

Origami-FA, O-FA Folic Acid adorned origami; H-O HSV1 wrapped in origami; H-O-FA HSV1 wrapped in folic acid-adorned origami.
