# Supplementary material for: DNA Origami Disguises Herpes Simplex Virus 1 Particles and Controls Their Virulence
Source: Molecules. 2022 Oct 23;27(21):7162. doi: 10.3390/molecules27217162 (PMC9656247; doi:10.3390/molecules27217162)
Supplement: Supplementary file 1 [file molecules-27-07162-s001.zip › molecules-1932178-supplementary.pdf]

Supplementary Information

DNA Origami Disguises Herpes Simplex Virus 1 Particles and Controls their Virulence

Raina M. Borum *, Avery E. Lin, Xiangyi Dong, Mingxuan Kai and Yi Chen

Department of NanoEngineering, University of California, San Diego, CA 92093, USA

* Correspondence:rborum@eng.ucsd.edu

1. **Supplementary Figures and Derivation**
2. **DNA Origami staple strand sequences**

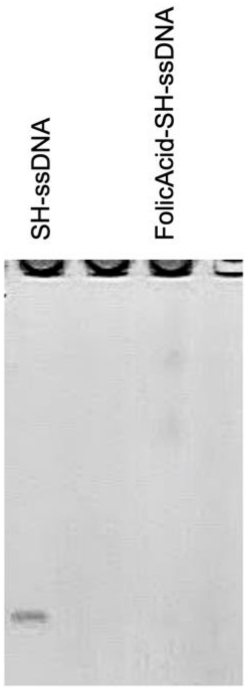

Figure S1. 20% Urea Denature PAGE on Folic Acid-DNA conjugation. When the thiolated ssDNA is covalently linked to Maleimide-PEG-Folic Acid as facilitated by TCEP reduction, the band for the thiolssDNA completely disappears. Both strands were loaded at identical starting concentrations.

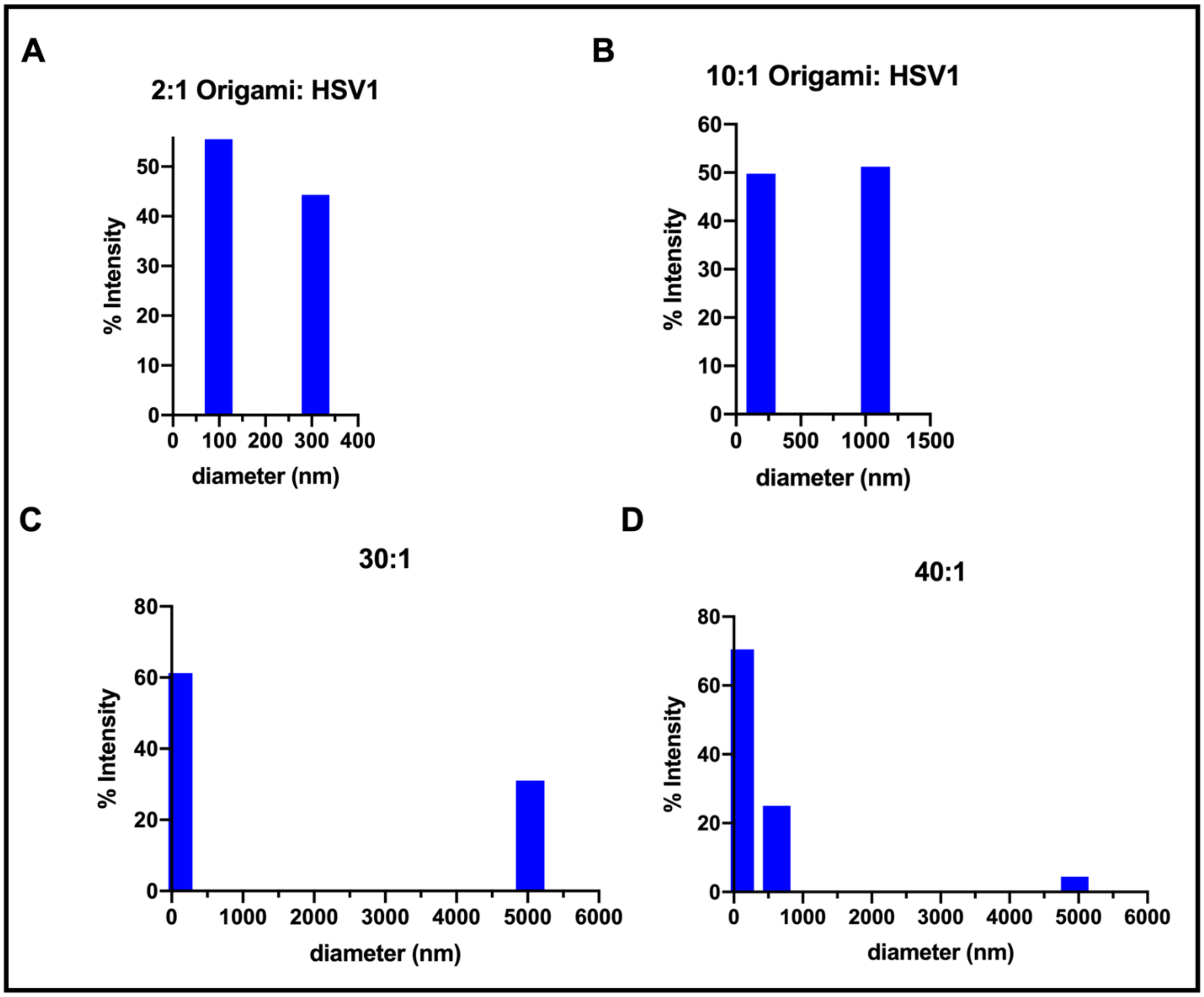

Figure S2. Supplementary DLS measurements of our origami:HSV1 complexes based on molar packing ratio. When origami:HSV1 molar ratios approached past 10:1 (191 nm, 1077 nm), and throughout 20:1 (119 nm, 4726 nm) and 30:1 (90 nm, 5036 nm), micron range agglomerates continued to grow in size, first developing from near 1000nm in size and approaching 5000nm in diameter at the maximum. As was otherwise characterized in our AGE and AFM experiments, we predicted a 40:1 ratio (90 nm, 622 nm, 4946 nm), being past the critical ratio, would indicate a sudden or noticeable change in these agglomerated sleeve behaviors.

Origami	(M13)	1	0	0.5	1	3	10	30	40
HSV1	0	0	1	1	1	1	1	1	1

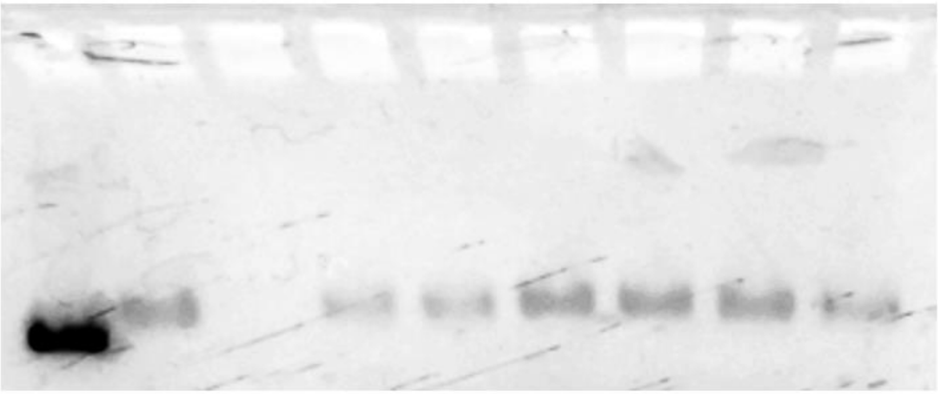

Figure S3. Further 1% AGE validation of Origami-HSV1 loading depending on molar ratios.

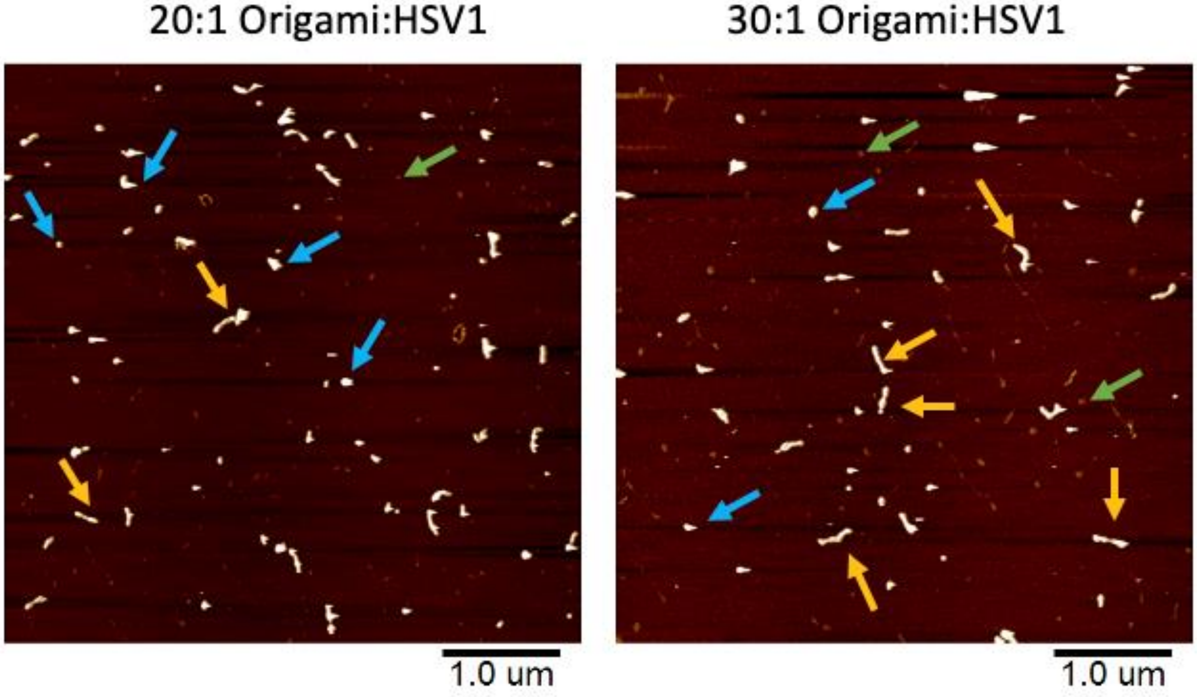

Figure S4. Low Magnification Atomic Force Micrographs of DNA Origami-HSV1 Complexes show a distribution of different complex geometries, ranging from pocket like structures (as indicated with blue arrows), sleeves (yellow arrows) and occasional unbound origami (green)

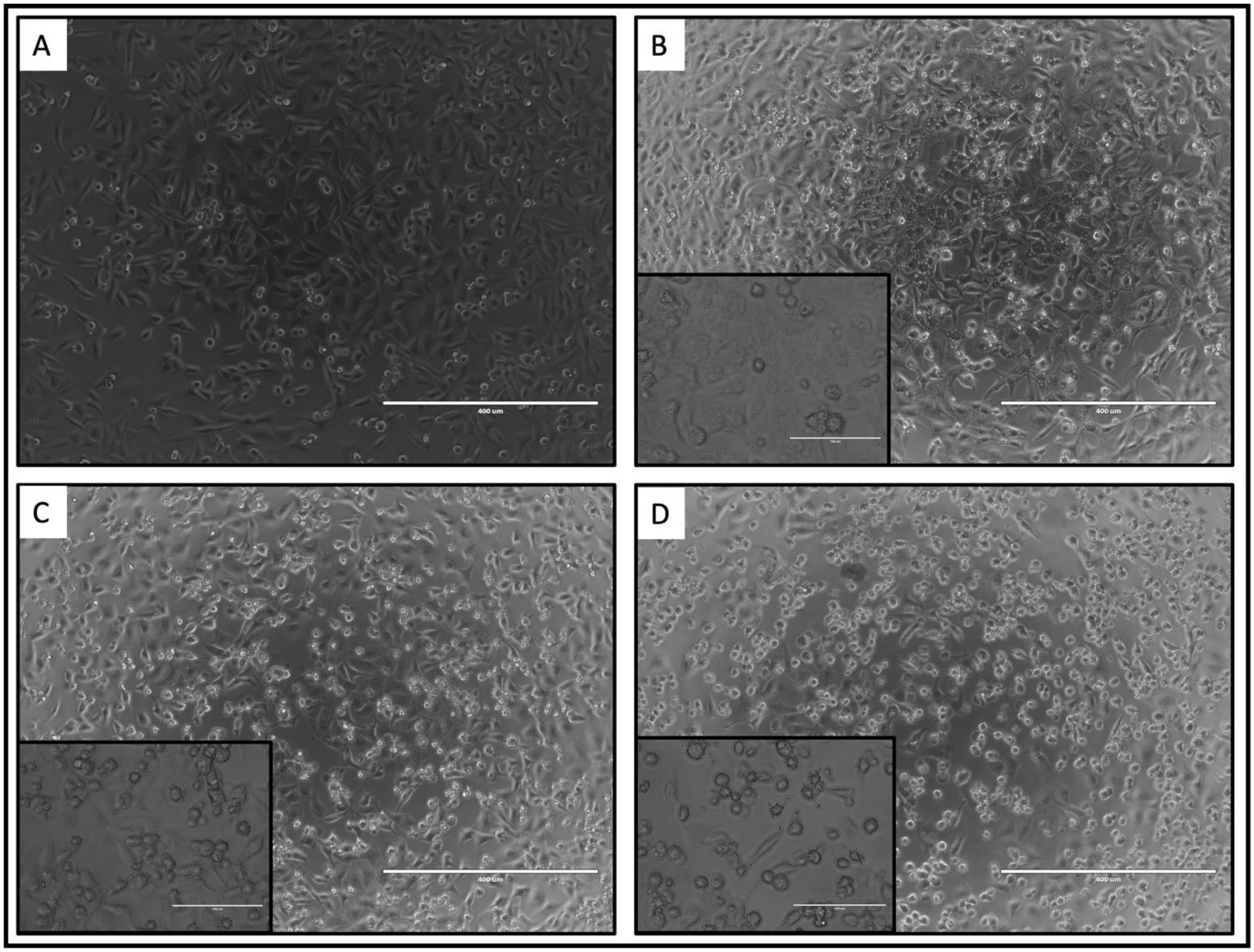
A
400 um
B
400 um
C
400 um
D
400 um

Figure S5. HeLa cells 24 hours post infection. **(A)** Mock-infected (control), **(B)** HSV1-infected, **(C)** HSV1-Origami infected, and **(D)** HSV1-Origami-Folate infected where most the cells were visibly detached**.**

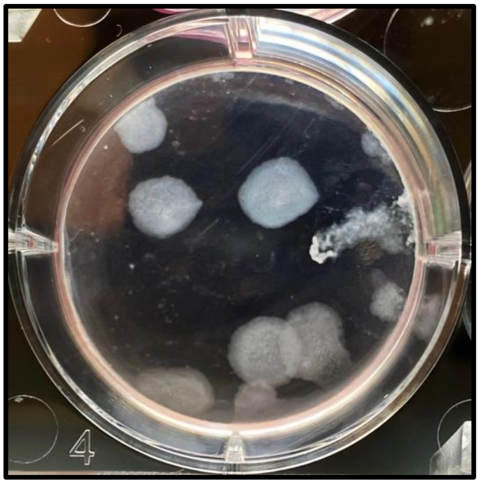

Figure S6. Plaque Assay result of HeLa inoculated with naked M13 strands at the same concentration as inoculated origami. This corresponds nicely to the same HeLa plaque assay in Figure 4.

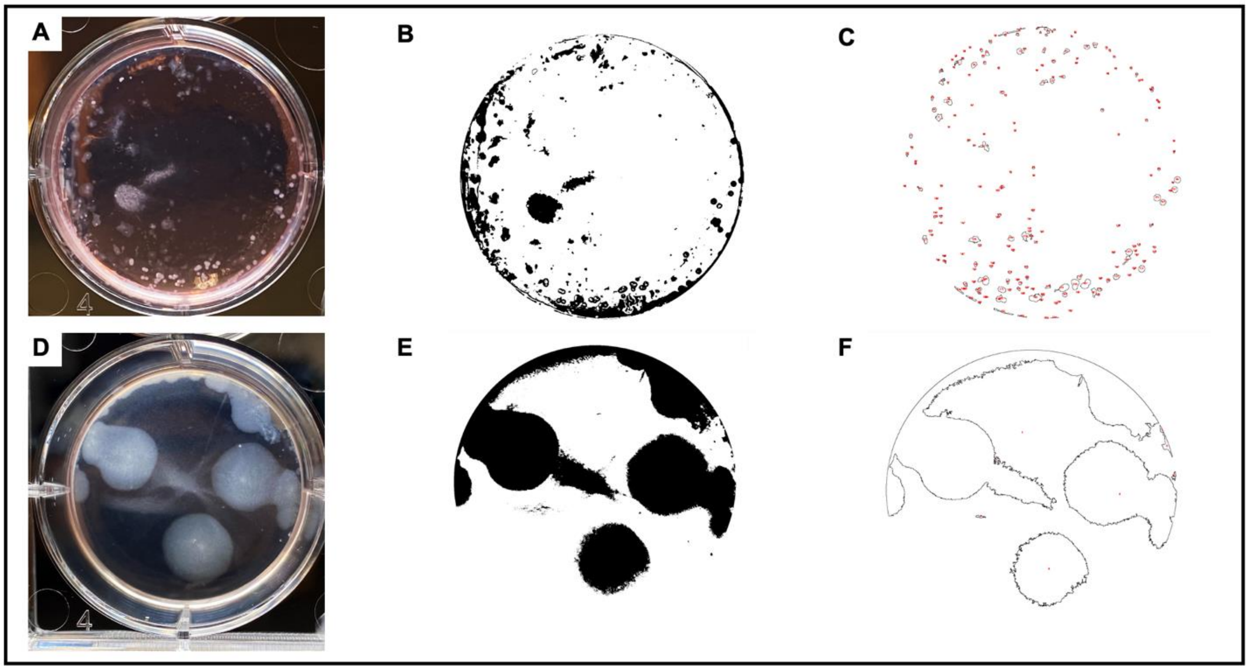

Figure S7. Plaque Assay Plaque counting using ImageJ. (**A,D**) original image of plaque in well. (**B,E**) Image after adjusted to 8-bit image type with threshold tuned to capture as many isolated plaques as possible. Threshold conditions were kept constant for all wells during plaque counting. (**C,F**) Resultant counted plaques when using Particle Analysis tool in ImageJ. **(A-C)** shows the B16F10 plaque assay post HSV1 infection, while **(D-F)** shows the same infection with HeLa. Both cell lines resulted in these characteristically different plaque morphologies.

Derivation of parallel function used for infectivity (PFU/mL) calculations for origami-HSV1 complex cases. Here, D_o is dilution factor for inoculated origami and D_H is dilution factor for inoculated HSV1. Plaque number (P) and total inoculum volume (V) are held constant as both origami and HSV1 are administered at the same time as a complex, resulting in one constant plaque number under the total volume of the inoculum. Therefore, it is appropriate to only analyze the adjusted infectivity calculation (PFU/mL) with respect to differing dilution factors for both entities.

$$\frac{PFU}{mL} = \frac{P}{D * V}$$

$$\frac{P}{D * V} = \frac{P}{D_O * V} + \frac{P}{D_H * V}$$

$$\frac{1}{D} = \frac{1}{D_O} + \frac{1}{D_H}$$

$$\frac{1}{D} = \frac{D_o + D_H}{D_O * D_H}$$

$$\therefore \boldsymbol{D} = \frac{\boldsymbol{D_O} * \boldsymbol{D_H}}{\boldsymbol{D_O} + \boldsymbol{D_H}}$$

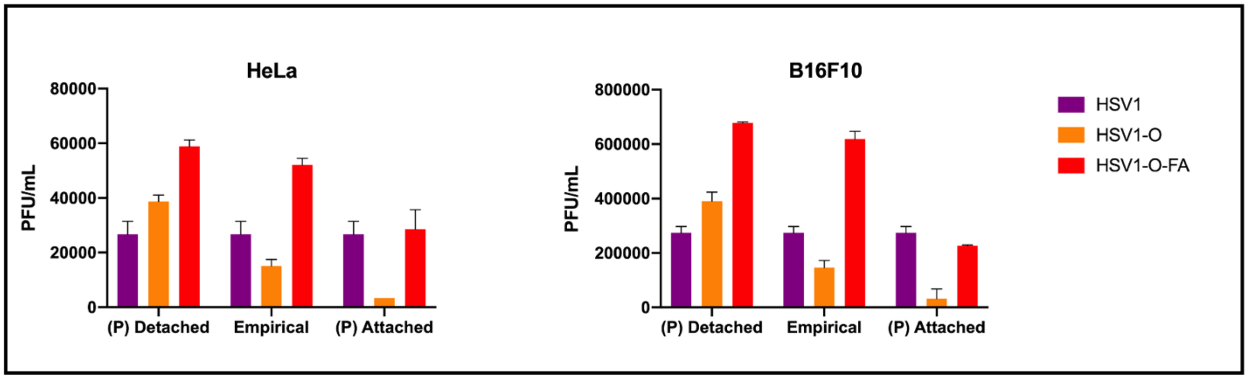

Figure S8. Predicted infectivity rates based on complex stability or instability in vitro, as compared with empirical results. We were primarily concerned that our plaque numbers resulted from full detachment of our complexes in vitro.

Staple strand sequences (scaffold strand: M13mp18)
GAGCCCCCGATTTAGAGCTTGACGGG
CAAGTTTTTTGGGGTCGAGGTGCCGT
CGAAAAACCGTCTATCAGGGCGATGG
AGTTTGGAACAAGAGTCCACTATTAA
AATCGGCAAAATCCCTTATAAATCAAAAGAATAG
CTGGGGTGCCTAATGAGTGAGCGTGGCGAGAAAGGAAGGGAA
GAAAGCCGGCGAACTAACTCACATTA
CCAGTCGGGAAACGGAACCCTAAAGG
AAAGCACTAAATCCTGTCGTGCCAGC
GGGAGAGGCGGTTCCATCACCCAAAT
CCCACTACGTGAATGCGTATTGGGCG
GAGACGGGCAACACAACGTCAAAGGG
AGAACGTGGACTCGCTGATTGCCCTT
GCAAGCGGTCCACTGAGTGTTGTTCC
CCCGAGATAGGGTGCTGGTTTGCCCC
ATTGCGTTGCGCTCTCACAATTCCAC
AAATTGTTATCCGCACTGCCCGCTTT
TGCATTAATGAATCTCGAATTCGTAA
CCCGGGTACCGAGCGGCCAACGCGCG
CCAGGGTGGTTTTTGCCAAGCTTGCA
AAACGACGGCCAGTCTTTTCACCAGT
CACCGCCTGGCCCGTTGGGTAACGCC
GCAAGGCGATTAATGAGAGAGTTGCA
AGCAGGCGAAAATTCTTCGCTATTAC
GAAGGGCGATCGGTGCGGGCCCCTGTTTGATGGTGGTTCCGA
CGCGTCTGGCCTTCCTGTAGCCGGAAGCATAAAGTGTAAAGC
ACAACATACGAGCCAGCTTTCATCAA
GTCGGATTCTCCGTGTTTCCTGTGTG
TCATGGTCATAGCTGGGAACAAACGG
CGTTGGTGTAGATACTCTAGAGGATC
TGCCTGCAGGTCGGGGCGCATCGTAA
GACGACAGTATCGTCACGACGTTGTA
AGGGTTTTCCCAGGCCTCAGGAAGAT
GCTTCTGGTGCCGAGGGGGATGTGCT
GCCAGCTGGCGAAGAAACCAGGCAAA
CATTAAATGTGAGTAAATCAGCTCAT
ATTAAATTTTTGTCGAGTAACAACCC
CGGATTGACCGTATATTTAAATTGTA
TTGTATAAGCAAAATGGGATAGGTCA
CCGTGCATCTGCCCCCCGGTTGATAA

TCAATCATATGTAAGTTTGAGGGGAC
CGCACTCCAGCCAACAAGAGAATCGA
GAGTCTGGAGCAAGCTTTCCGGCACC
GCGCCATTCGCCATTTTGAGAGATCT
AATGCCGGAGAGGGTAGCTATTTCAGGCTGCGCAACTGTTGG
CAATAAATCATACAGGCAAGGGGAACGCCATCAAAAATAATT
TTTTTAACCAATACAAAGAATTAGCA
TAAAGCTAAATCGTGTTAAAATTCGC
AACGTTAATATTTGTTGTACCAAAAA
GGAGAAGCCTTTAAAAAACAGGAAGA
TCAGAAAAGCCCCTTTCAACGCAAGG
TTTTAAATGCAATTAAAACTAGCATG
TGAACGGTAATCGGCCTGAGTAATGT
AAGGCCGGAGACAGGTCATTGCCTGA
ACAAAGGCTATCAGTCAAATCACCAT
AAATTAAGCAATAGCTGAAAAGGTGG
ATTTGGGGCGCGAAAGCCTCAGAGCA
CATTATGACCCTGATTTCGCAAATGG
GACCATTAGATACTAATACTTTTGCG
ATAAAAATTTTTACAGTTGATTCCCA
TCATTCCATATAAGAACCCTCATATA
GTAGGTAAAGATTTGTTTTAAATATG
CTGTAGCTCAACACAAAAGGGTGAGA
CAATATGATATTCTTTTGCGGATGGC
CTCCTTTTGATAAGAGGTCATAACCGTTCTAGCTGATAAATT
ATGTTTAGACTGGATAGCGTCAATAGTAGTAGCATTAACATC
CATCAATTCTACTCAATACTGCGGAA
CTCAAATGCTTTATAGCTATATTTTC
TCAATAACCTGTTAACAGTTCAGAAA
AGGTCTTTACCCTGTAGATTTAGTTT
ATTCTGCGAACGAGACTATTATAGTC
GATTAAGAGGAAGGTGTCTGGAAGTT
CAACTAAAGTACGCCCGAAAGACTTC
AAAGCGAACCAGAGCTGAATATAATG
TTAGAGCTTAATTCCGGAAGCAAACT
TCGTCATAAATATCGAGAGGCTTTTG
AAAACCAAAATAGTCATTGAATCCCC
ACGAGAATGACCAAGCAACACTATCA
GAGGCATAGTAAGTAAATCAAAAATC
AGAAGCAAAGCGGATTCAACTAATGC
TTAGGAATACCACATTGCATCAAAAA

AAATATCGCGTTTACAACATTATTAC
ACGAACTAACGGATAATTCGAGCTTC
CCAACAGGTCAGGCCAGTCAGGACGT
TTTAAGAACTGGCTCATTATAATTAGAGAGTACCTTTAATTG
CCGGAACGAGGCGCAGACGGTGCCAGAGGGGGTAATAGTAAA
CAAAAGAAGTTTTCAATCATAAGGGA
ACAGATGAACGGTCCAGACGACGATA
TAACCCTCGTTTAGTACAGACCAGGC
AGTAATCTTGACACAAAAGGAATTAC
AGATACATAACGCAGAACCGGATATT
TGCTCATTCAGTGCATCAGTTGAGAT
AGGTAGAAAGATTAATAAGGCTTGCC
TAAATTGGGCTTGCTACGTTAATAAA
TGGGAAGAAAAATAGATGGTTTAATT
ACCGAACTGACCAGCCTGATAAATTG
GATTTGTATCATCACTTTGAAAGAGG
GCATAGGCTGGCTCCCAGCGATTATA
CTCATCTTTGACCGACCTTCATCAAG
CATTACCCAAATCCAACCTAAAACGA
ACTACGAAGGCACAACGTAACAAAGC
CTGACGAGAAACATGAGGAAGTTTCC
AAAGACTTTTTCACCAGAACGAGTAG
TCAACTTTAATCAGGAACGAGGGTAG
TCAGCAGCGAAAGACAGCATCTTGTGAATTACCTTATGCGAT
AACTTTCAACAGTTTCAGCGGGACCTGCTCCATGTTACTTAG
TGTCGAAATCCGCAGTGAGAATAGAA
ATAATTTTTTCACAAAGTACAACGGA
CCAAGCGCGAAACGTTGAAAATCTCC
TAATTGTATCGGTATACACTAAAACA
AAGAGGCAAAAGATTATCAGCTTGCT
GATACCGATAGTTAATACGTAATGCC
ATTAAACGGGTAAGCGCCGACAATGA
ATATATTCGGTCGGGCTTTGAGGACT
CAACGGCTACAGACTGAGGCTTGCAG
AGGAACAACTAAATTTCCAGACGTTA
AAGTTTTGTCGTCGGAATTGCGAATA
AAAAAAAAGGCTCCATTCCACAGACA
ACAACGCCTGTAGCAAAAGGAGCCTT
TTCGAGGTGAATTCATGTACCGTAAC
CCCAATAGGAACCTCTTAAACAGCTT
CAACAACCATCGCACCCTCAGAGCCA

CCTCAGAACCGCCCCACGCATAACCG
GGAGTTAAAGGCCACTCAGGAGGTTT
CCGGAATAGGTGTATCACCGTGCTTTTGCGGGATCGTCACCC
GGAAAGCGCAGTCTCTGAATTCTGTATGGGATTTTGCTAAAC
GTAAATGAATTTTTACCGTTCCAGTA
CAGGAGTGTACTGGCGTAACGATCTA
GCCCTCATAGTTAGTAATAAGTTTTA
GCCCGTATAAACAACCAGTACAAACT
ACTGAGTTTCGTCGTTAATGCCCCCT
AACATGAAAGTATCAGGGATAGCAAG
CCACCCTCATTTTTAAGAGGCTGAGA
CGGGGTTTTGCTCTCAGAACCGCCAC
AGTACCGCCACCCAGTACCAGGCGGA
AGCGTCATACATGTTGGCCTTGATAT
GCAGGTCAGACGAGCTTTTGATGATA
ACGGGGTCAGTGCCACCACCAGAGCC
CCGCCACCAGAACCTTGAGTAACAGT
GCCTATTTCGGAACCCTCAGAACCGC
CTCAGAGCCGCCACCTATTATTCTGA
CTCCTCAAGAGAAAAAATCACCGGAA
TCTTTTCATAATCGGATTAGGATTAG
TAAGTGCCGTCGACGGCATTTTCGGT
CAGACTGTAGCGCGTTTTCATGAGGGTTGATATAAGTATAGC
TTATTTTGTCACAATCAATAGAATCCTCATTAAAGCCAGAAT
TCACAAACAAATAAAAATTCATATGG
GACATTCAACCGAACAGGAGGTTGAG
GCCGCCAGCATTGTTGAGGGAGGGAA
TAAAGGTGAATTAACCACCCTCAGAG
CACCCTCAGAGCCTCACCGTCACCGA
GCAAAATCACCAGCGGAACCGCCTCC
CCAGAGCCACCACTAGCACCATTACC
TGAAACCATCGATTTAGCGTTTGCCA
CATAGCCCCCTTAAGCAGCACCGTAA
TTTACCAGCGCCAAAAGGTGGCAACA
AAAATACATACATAAGACAAAAGGGC
GGTAAATATTGACATTAAGACTCCTT
AAGAACTGGCATGGGAAATTATTCAT
CTTGAGCCATTTGAAGGAAACCGAGG
ACAAAGTTACCAGGGAATTAGAGCCA
ATTAGCAAGGCCGTACCGAAGCCCTT
GCAATAGCTATCTGAAACGTCACCAA

TCAGTAGCGACAGGAGTTAAGCCCAA
GAGAGATAACCCACAAGAATTAATCAAGTTTGCCTTTAGCGT
AATCAAGATTAGTTGCTATTTCAAAGACACCACGGAATAAGT
TATAAAAGAAACGTGCACCCAGCTAC
TAACGAGCGTCTTTTAGCAAACGTAG
ATTACGCAGTATGTCCAGAGCCTAAT
ATTATTTATCCCAACGGAATACCCAA
AAACGCAATAATAATCCAAATAAGAA
GAAAATAGCAGCCAGCAGATAGCCGA
TTTAAGAAAAGTATTTACAGAGAGAA
GACGGGAGAATTAAAACAATGAAATA
TAATAAGAGCAAGACTGAACACCCTG
AATTTTATCCTGAAGGCGTTTTAGCG
TTCTAAGAACGCGATCTTACCAACGC
TTGCCAGTTACAACAATAGCAAGCAA
TCATTACCGCGCCAATAAACAGCCAT
ACGATTTTTTGTTTCGAGAACAAGCA
AGTACCGCACTCATAACGTCAAAAAT
TAACATAAAAACACTGTCTTTCCTTA
AATCAATAATCGGGGGAAGCGCATTA
AACAAAGTCAGAGAAATAATATCCCA
TAGATAAGTCCTGAACAAGAAGGTAATTGAGCGCTAATATCA
AATAAGAATAAACACCGGAATGCGGGAGGTTTTGAAGCCTTA
AACCTCCCGACTTCATAATTACTAGA
TATACAAATTCTTGGCTTATCCGGTA
ATCAGATATAGAAACCAGTATAAAGC
AGAATCGCCATATTTCATCGTAGGAA
AGCCGTTTTTATTTTAACAACGCCAA
GAGCCAGTAATAAGGGTATTAAACCA
TCATTCCAAGAACGAGAATATAAAGT
CCAGACGACGACAGCATGTAGAAACC
TCCTAATTTACGAATAAACAACATGT
AAAAGCCTGTTTATAAATTTAATGGT
TCATCTTCTGACCGTATCATATGCGT
CAACGCTCAACAGGAACGCGAGAAAA
ATCGCAAGACAAATAGGGCTTAATTG
CATGTAATTTAGGTGGGTTATATAAC
CTCCGGCTTAGGTCAGAGGCATTTTC
ACCGACAAAAGGTTTTATCAAAATCA
AGTCAATAGTGAAAAAGTAATTCTGT
TCAGCTAATGCAGTGAAAACATAGCG

TTAATTTTCCCTTAGAATCCTAACGCGCCTGTTTATCAACAA
TTTAACGTCAGATGAATATACCGTGTGATAAATAAGGCGTTA
TTGAAATACCGACAGTAACAGTACCT
TTCGCCTGATTGCATTTTAGTTAATT
CTTTTTCAAATATTTTGAATACCAAG
TTCATTTCAATTATGATGCAAATCCA
TATATGTAAATGCCCTGAGCAAAAGA
CAAAATTAATTACCTACCTTTTTAAC
TAGGTCTGAGAGAATTTAACAATTTC
CAGTACATAAATCAGACGCTGAGAAG
ATAGCTTAGATTAAATATATGTGAGT
TTTACATCGGGAGTTATTTGCACGTA
TACCATATCAAAAAAACAATAACGGA
TTACAAAATCGCGGTTTGGATTATAC
TATAATCCTGATTCAGAGGCGAATTA
AGATGATGAAACACATCATATTCCTG
GGAGCGGAATTATAACATCAAGAAAA
ATTTGAATTACCTGTAACATTATCAT
TTTAAAAGTTTGATTTTTAATGGAAA
GAATAACCTTGCTGACAACTCGTATT
ATTAGACTTTACAAACAATTCTCTGTAAATCGTCGCTATTAA
AATACCGAACGAACCACCAGCGAAATTGCGTAGATTTTCAGG
AAACAGAAATAAAAGAAGATAAAACA
GCCTGCAACAGTGAAGGGTTAGAACC
TTCTGAATAATGGCCACGCTGAGAGC
TCACCTTGCTGAAGGCAATTCATCAA
ATTATCAGATGATCCTCAAATATCAA
GCAAATCAACAGTGAAACCACCAGAA
TTTGCGGAACAAATGAAAGGAATTGA
AGCACTAACAACTGAACGTTATTAAT
AAATCCTTTGCCCAATAGATTAGAGC
GAGGTGAGGCGGTTTAGTCTTTAATG
TTTTGAATGGCTACAGTATTAACACC
CAGCAGCAAATGACTGACCTGAAAGC
AGATAGAACCCTTAAAATCTAAAGCA
ACCCTCAATCAATTCACACGACCAGT
GCAGATTCACCAGATCTGGTCAGTTG
GGAAGGTTATCTATTTTGACGCTCAA
GGAAATACCTACAAAATATCTTTAGG
CGTCAATAGATAAAATATTACCGCCA
CTTGCTGGTAATATCCAGAACTACATTTGAGGATTTAGAAGT

GCGCTTAATGCGCCGCTACAGCCCTAAAACATCGCCATTAAA
CGCGAACTGATAGGGCGCGTACTATG
GCTTTCCTCGTTAGCACAGACAATAT
GTAAGAATACGTGGAATCAGAGCGGG
ATTTTAGACAGGATTCTGGCCAACAG
AATAAAAGGGACAACGGTACGCCAGA
TGAGGCCACCGAGATTATTTACATTG
TCGTCTGAAATGGTAAAAGAGTCTGT
AATACTTCTTTGAGAAAAACGCTCAT
GCCATTGCAACAGTTAGTAATAACAT
GTTGCTTTGACGAGCACGTATAACGT
AGCTAAACAGGAGGCCGATTAAAGGG
ATCCTGAGAAGTGTTTTTATAATCAG
CCATCACGCAAATTAACCGTTGTAGC
CACTTGCCTGAGTAGAAGAACTCAAACTATCGGC
